# Supplementary material for: Elective Surgery in Adult Patients with Excess Weight: Can Preoperative Dietary Interventions Improve Surgical Outcomes? A Systematic Review
Source: Nutrients. 2021 Oct 25;13(11):3775. doi: 10.3390/nu13113775 (PMC8623302; doi:10.3390/nu13113775)
Supplement: Supplementary file 1 [file nutrients-13-03775-s001.zip › nutrients-1394711-supplementary.pdf]

Study or Subgroup	Preop diet Mean [minutes]	Preop diet SD [minutes]	Preop diet Total	Control Mean [minutes]	Control SD [minutes]	Control Total	Weight	Mean Difference IV, Random, 95% CI	A	B	C	D	E	F	G	H	I	J	K
Burnand 2016 (lap chole, VLCD)	27.25	5.75	21	63	37.5	25	23.1%	-35.75 [-50.65, -20.85]	+	+	+	+	+	+	+	+	+	+	+
Hollis 2020 (various gen surg, VLCD)	89.9	29.3	23	107.5	41.4	23	19.5%	-17.60 [-38.33, 3.13]	+	+	+	+	+	+	+	+	+	+	+
Barth 2019 (liver resection, VLCD)	246	72	30	258	90	30	10.0%	-12.00 [-53.24, 29.24]	+	+	+	+	+	+	+	+	+	+	+
de Luis 2012 (joint replacement, LCD)	86.5	21.8	20	91.8	41.7	20	19.6%	-5.30 [-25.92, 15.32]	+	−	?	?	−	+	+	?	+	?	?
Inoue 2019 (gastrectomy, VLCD*)	368.25	92.75	27	372	56	23	9.8%	-3.75 [-45.56, 38.06]	+	−	?	−	?	+	+	+	+	+	?
Liang 2018 (hernia repair, general healthy eating)	102.3	55.8	44	91	49.2	34	18.0%	11.30 [-12.05, 34.65]	+	+	−	+	+	+	+	+	+	+	?
**Total (95% CI)**			**165**			**155**	**100.0%**	**-12.27 [-28.42, 3.87]**											

Heterogeneity: Tau² = 235.60; Chi² = 13.40, df = 5 (P = 0.02); I² = 63%
Test for overall effect: Z = 1.49 (P = 0.14)

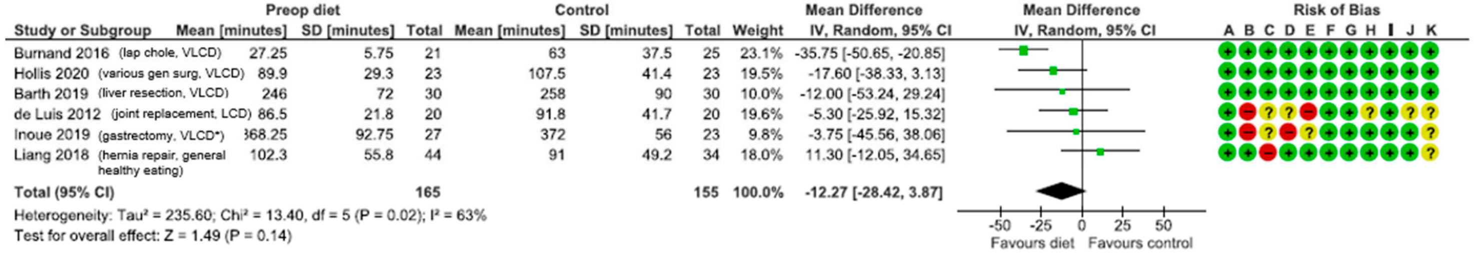

Risk of bias legend
(A) Clear research question
(B) No selection bias
(C) Comparable groups
(D) Handling of withdrawrals
(E) Blinding used
(F) Interventions described
(G) Outcomes and measurements valid
(H) Statistical analysis appropriate
(I) Conclusions supported and biases considered
(J) Funding / sponsorship bias unlikely
(K) Overall

*although described as 'VLCD', was likely >900kcal

Supplementary Figure S1. Forest plot of operating time outcome for studies that gave preoperative dietary intervention and compared time between groups
